# Factors Associated with Complications in Pediatric Foreign Body Ingestion: A Retrospective Cohort Study of 754 Children

**DOI:** 10.3390/children13101381

**Published:** 2026-10-12

**Authors:** Gülser Esen Besli, Sibel Hatice Özümüt, Nazira Amiraslanova, Çiğdem Ulukaya Durakbaşa

**Affiliations:** 1 Department of Pediatric Emergency Medicine, Faculty of Medicine, Göztepe Prof. Dr. Süleyman Yalçın City Hospital, Istanbul Medeniyet University, 34722 Istanbul, Türkiye; 2 Department of Pediatrics, Göztepe Prof. Dr. Süleyman Yalçın City Hospital, Istanbul Medeniyet University, 34722 Istanbul, Türkiye; sibel.ozumut@medeniyet.edu.tr (S.H.Ö.); nazira.amiraslanova@ogu.edu.tr (N.A.); 3 Department of Pediatric Surgery, Faculty of Medicine, Istanbul Medeniyet University, 34722 Istanbul, Türkiye; cigdem.durakbasa@medeniyet.edu.tr

**Keywords:** foreign body ingestion, child, complications, button battery, magnets, esophageal foreign body, pediatric emergency

## Abstract

**Highlights:**

**What are the main findings?**

- In a cohort of 754 children, most ingested foreign bodies were asymptomatic and passed spontaneously (75.9%); complications were uncommon (3.8%), with major complications occurring in only 1.2%. Vomiting, sharp or pointed objects, and pharyngeal or esophageal location were associated with complications in a parsimonious multivariable model.
- Coins had a relatively high removal rate, particularly when located in the esophagus, but rarely caused harm, whereas button batteries accounted for most major complications. Major complications occurred with esophageal button batteries and with multiple magnets and sharp objects located in the stomach or intestine.

**What are the implications of the main findings?**

- These simple warning signs may help emergency physicians identify children who warrant closer evaluation and prioritize specialist assessment; however, they have not been validated as a clinical decision rule.
- The risk of serious harm depends not only on the need for removal but also on the intrinsic hazard of the object and its anatomical location. Esophageal button batteries require immediate removal, while multiple magnets and sharp or pointed objects warrant urgent assessment and appropriate intervention or close monitoring.

**Abstract:**

**Background/Objectives**: Foreign body (FB) ingestion is a common reason for pediatric emergency department visits. Although most ingested FBs pass spontaneously, a minority cause serious complications, and rapid identification of high-risk ingestions is crucial. We aimed to describe the management strategies and to identify clinical and FB-related factors associated with complications. **Methods**: We retrospectively reviewed 754 children aged <18 years who presented to a tertiary pediatric emergency department with FB ingestion between 2016 and 2022. FB types, anatomical locations, clinical findings, interventions, and complications were analyzed. Univariate logistic regression was used to screen candidate variables, followed by a parsimonious multivariable model limited to a single non-redundant variable per clinical domain to identify factors independently associated with complications. **Results**: Of the 754 children, 434 (57.6%) were male, with a median age of 3.6 years. Coins were the most commonly ingested objects (41.8%). Spontaneous passage occurred in 572 (75.9%) cases, while 182 (24.1%) required intervention, most commonly endoscopy (18.0%). Complications occurred in 29 children (3.8%), including 9 (1.2%) with major complications. Major complications occurred almost exclusively with button batteries in the upper esophagus and with multiple magnets and sharp objects in the stomach or intestines. Although intervention was required in 22.5% of coin ingestions, the complication rate was only 0.3%. In multivariable analysis, vomiting (odds ratio [OR], 5.65), sharp or pointed objects (OR, 6.34), and pharyngeal or esophageal location (OR, 12.57) were independently associated with complications. **Conclusions**: These interrelated features may help emergency physicians identify children who need closer evaluation, but they require prospective validation before being used for clinical prediction.

## 1. Introduction

Foreign body (FB) ingestion is one of the most frequent reasons for emergency department (ED) presentation in the pediatric age group, particularly in children between 6 months and 3 years of age, for whom exploring the environment orally is a normal part of development [1,2]. Most ingested FBs pass through the gastrointestinal (GI) tract uneventfully and are expelled spontaneously without any intervention [3,4]. Spontaneous passage rates reported in previous pediatric series are high, commonly ranging from approximately 60% to 90% [4,5], and conservative outpatient management is therefore suitable for many children.

However, a clinically significant minority of ingestions may result in serious, occasionally life-threatening complications, including esophageal burn, perforation, fistula formation, mediastinitis, or stricture, and intestinal obstruction or perforation [6,7]. The risk of complications is strongly influenced by both the type of object and its anatomical location. Esophageal FBs and high-risk objects, including button batteries, multiple magnets, and sharp or pointed objects, generally require urgent evaluation and removal [8,9,10]. Current guidelines from the European Society for Paediatric Gastroenterology, Hepatology and Nutrition (ESPGHAN) and the North American Society for Pediatric Gastroenterology, Hepatology, and Nutrition (NASPGHAN) provide a framework for risk stratification and the timing of intervention [11,12].

From the perspective of emergency physicians, the principal challenge is to rapidly identify the small proportion of children who require urgent endoscopic or surgical intervention while avoiding unnecessary procedures in those who can be safely observed. Although several studies have investigated the clinical characteristics, management, and outcomes of pediatric FB ingestion, substantial variation remains in the reported spectrum of ingested objects, indications for intervention, and rates of complications [13,14,15,16,17]. Recent large pediatric cohorts have provided valuable data on factors associated with endoscopic intervention [4,14]; however, further characterization of the relationship between the initial clinical presentation, FB characteristics, management decisions, and subsequent complications could help improve early risk stratification in emergency departments.

In this study, we evaluated a large single-center pediatric cohort presenting with suspected FB ingestion. We aimed to describe the management strategies applied according to FB characteristics and the child’s clinical condition; to determine the frequency and severity of complications; and to identify the clinical and FB-related factors associated with complications.

## 2. Materials and Methods

### 2.1. Study Design and Population

**Figure 1:**
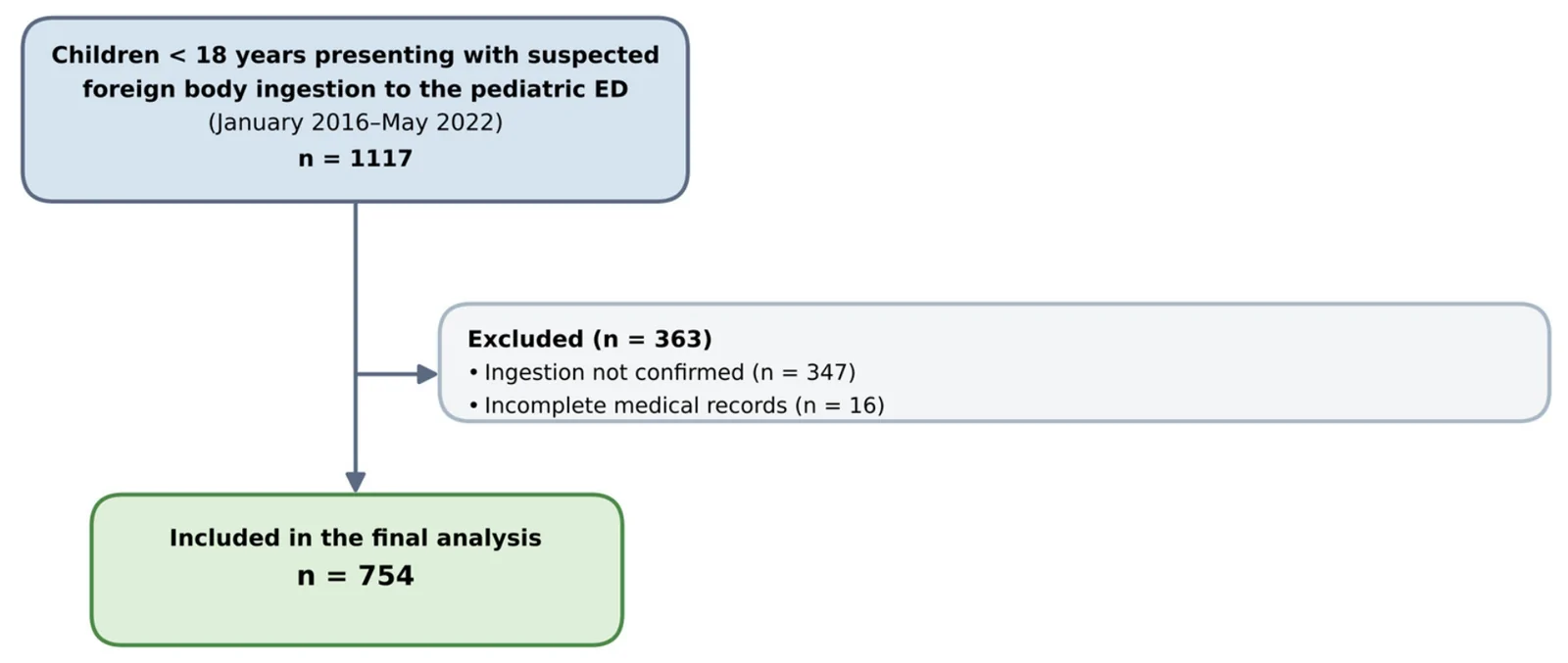
The study flowchart.

This was a retrospective, single-center observational study conducted in the pediatric ED of a tertiary referral hospital. Between January 2016 and May 2022, all children under the age of 18 who presented with suspected FB ingestion were assessed for eligibility. We included children with a confirmed ingestion and complete medical records; 754 children constituted the final study population. The study flowchart is shown in Figure 1. The study was conducted in accordance with the Declaration of Helsinki and approved by the institution’s ethics committee (IRB No. 2022/0308, dated 18 May 2022). Due to the retrospective design, the requirement for informed consent was waived. The STROBE (Strengthening the Reporting of Observational Studies in Epidemiology) reporting guideline and checklist were used in the preparation of this article.

### 2.2. Data Collection

For each patient, demographic characteristics (age, sex, comorbidities), type and anatomical location of the FB, clinical signs and symptoms at presentation, interventional treatments, and complications were extracted from electronic medical records (Nucleus Medical Information System, Version 9.2.40, Monad Software and Consultancy Inc., Ankara, Türkiye) and procedure reports. To identify delayed complications, the electronic records of all children were reviewed for re-presentation to our institution and subsequent outcomes for at least 30 days after presentation. Foreign bodies were classified according to their type (coin, sharp/pointed object, battery, magnet, food bolus, or other) and anatomical location (pharynx, esophagus, stomach, duodenum, jejunum/ileum, or colon). Foreign body ingestion was confirmed by plain radiography (anteroposterior chest and/or abdominal radiographs, with lateral neck radiographs when a pharyngeal or cervical esophageal object was suspected), by direct visualization on oropharyngeal examination, or during endoscopic or Magill forceps extraction. Radiolucent ingestions with high clinical suspicion underwent oropharyngeal and/or esophagoscopic examination. No child underwent standard contrast studies, ultrasonography, or computed tomography to confirm ingestion.

### 2.3. Definitions and Outcomes

Complications were defined as mucosal or transmural injury directly attributable to the FB itself or iatrogenic damage resulting from the removal procedure, diagnosed endoscopically, surgically, or radiologically. Complications were classified as those present before any intervention (caused by the FB itself), those caused by the removal procedure (iatrogenic), and those developing during hospitalization or follow-up. Failure of spontaneous passage and the need for intervention were treated as separate outcomes and were not considered complications. Minor complications comprised local mucosal injury (mucosal hyperemia, edema, erosion, or bleeding). Impaction or mucosal penetration of an object without accompanying mucosal injury was regarded as an indication for removal rather than a complication. Major complications were defined as potentially life-threatening events, such as esophageal burn, stricture, perforation, fistula, mediastinitis, necrosis, gastrointestinal hemorrhage, or intestinal obstruction, adhesion, or perforation. Spontaneous passage was defined as expulsion of the FB without any interventional procedure. Interventional procedures comprised endoscopic removal, Magill forceps extraction, Foley catheter retrieval, and laparotomy.

### 2.4. Statistical Analysis

Statistical analyses were performed using SPSS version 24.0 (IBM Corp., Armonk, NY, USA). Penalized (Firth) logistic regression and bootstrap validation were performed in R version 4.3.3 (R Foundation for Statistical Computing, Vienna, Austria) using the logistf (version 1.26.0) and pROC (version 1.18.5) packages. The Shapiro–Wilk test was used to assess the distribution of continuous variables; non-normally distributed variables are presented as median (interquartile range), and categorical variables are presented as number (percentage). The Mann–Whitney U test was used to compare non-normally distributed parameters, and the chi-squared test was used to compare categorical data. We used univariate binary logistic regression to screen candidate variables associated with complication development, and we calculated 95% confidence intervals (CI) and odds ratio (OR) values. A two-sided *p*-value < 0.05 was considered statistically significant. All analyses were performed on cases with complete data; no imputation was undertaken for missing data.

Because complications were relatively rare (29 events), the multivariable model was deliberately kept parsimonious according to a domain-based strategy defined on clinical grounds to avoid overfitting: it was limited to a single non-redundant variable from three clinically relevant domains (presenting symptom, type of FB, and anatomical location) to obtain approximately ten outcome events for each variable. For the symptom domain, vomiting was selected as the representative marker. Dysphagia/odynophagia, hypersalivation, and gagging/feeding refusal are direct local manifestations of a pharyngeal or esophageal foreign body and were strongly associated with an upper-GI-tract location (e.g., 87% of children with dysphagia/odynophagia had a pharyngeal/esophageal foreign body; Phi (φ) = 0.58). Therefore, including these alongside anatomical location would have led to unnecessary repetition. In contrast, abdominal pain was almost entirely associated with intestinal location and occurred in only 22 children (4 of whom had complications), preventing a stable estimate. Vomiting was the least dependent symptom on the location of the foreign body (φ = 0.19), yet it occurred frequently enough and was independently associated with complications; therefore, it was retained as a non-redundant symptom marker. In a sensitivity analysis, dysphagia/odynophagia was substituted for vomiting to assess whether the location estimate was robust to the choice of symptom marker.

Among FB types, the sharp/pointed category was chosen as the representative high-risk object because it was both clinically intervenable and statistically stable (135 children, 16 complications). Although food-bolus impaction showed a high OR in the univariate analysis, this was calculated based on only 8 children (3 with complications) and gave an unstable estimate that changed direction when included in the multivariable model. Therefore, it was not kept as a covariate. For the location domain, pharyngeal and esophageal cases (pharynx and upper, middle, and lower esophagus; *n* = 180) were combined into a single upper-gastrointestinal-tract variable. The binary “symptomatic” variable was not included in the multivariable model due to near-complete discrimination (28 out of 29 complications occurred in symptomatic children); this finding was instead reported descriptively and in a univariate analysis. Collinearity was assessed using variance inflation factors; a penalized (Firth) logistic regression was additionally fitted given the low event count; model calibration was assessed with the Hosmer–Lemeshow test and discrimination with the c-statistic; and internal validation was performed by bootstrap resampling (1000 replicates).

### 2.5. Use of Generative AI and AI-Assisted Technologies in the Writing Process

During the preparation of the manuscript, Claude (Claude Opus 5.5 version, Anthropic PBC, San Francisco, CA, USA) and ChatGPT (GPT–4o version, OpenAI, San Francisco, CA, USA) were used to assist with language editing, grammar correction, formatting, and the preparation of figures. All AI-assisted content was reviewed and verified by the authors, who take full responsibility for the accuracy and integrity of the final manuscript.

## 3. Results

### 3.1. Demographic and Foreign Body Characteristics

A total of 754 children were analyzed; 434 of them (57.6%) were male. The median age was 3.6 years (interquartile range 2.2–5.7), and most patients (534; 70.8%) were between 1 and 5 years old. Twenty-one children (2.8%) had a comorbid condition; the most common were neuromotor disorder (11; 1.5%), behavioral disorder (6; 0.8%), or anatomical abnormality (4; 0.5%). The most common ingested objects were coins (315; 41.8%). In terms of anatomical location, 180 objects (23.9%) were found in the pharynx or esophagus, while the remainder reached the stomach or beyond (Table 1).

### 3.2. Clinical Presentation, Management, and Outcomes

**Figure 2:**
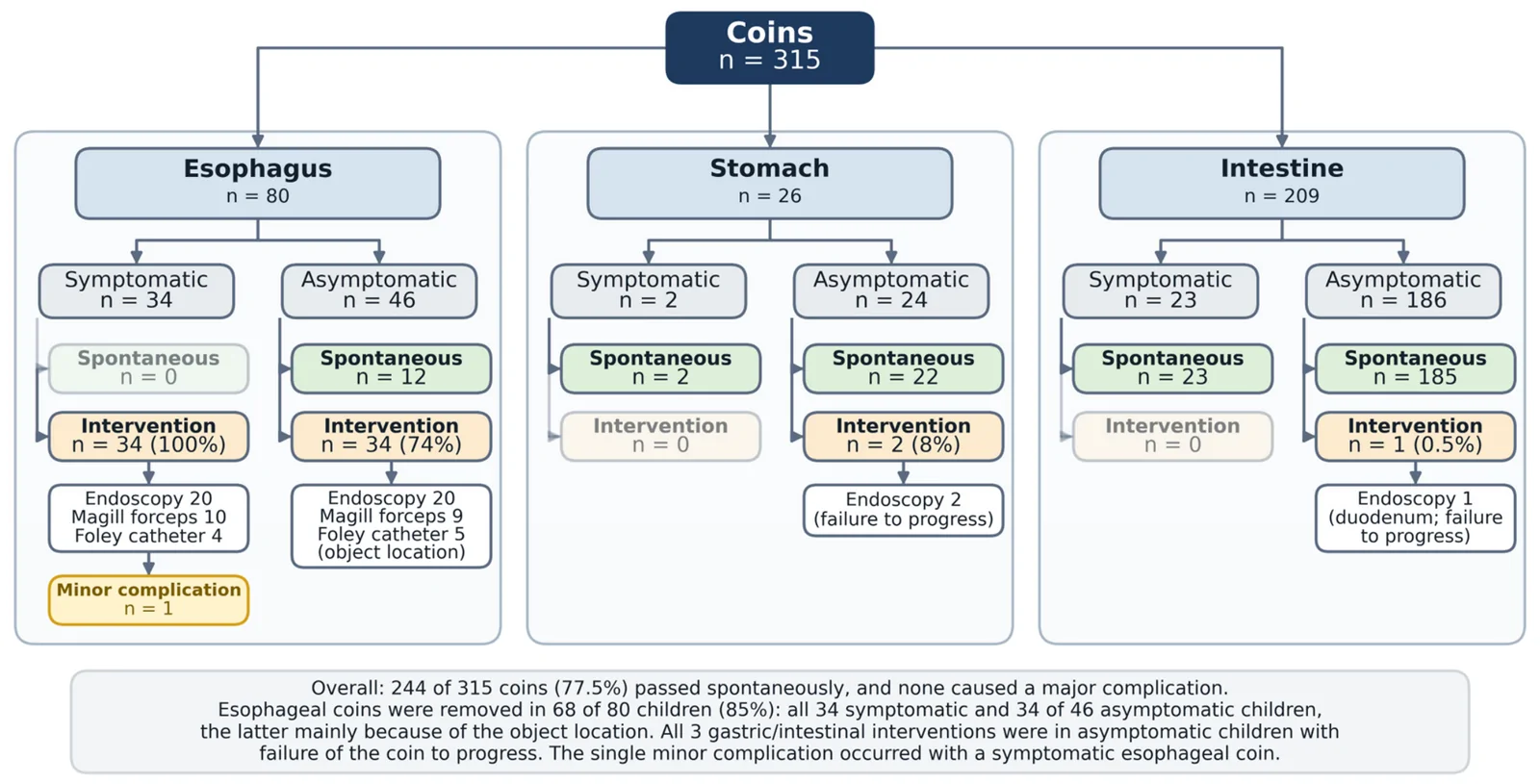
Management approach and outcomes for ingested coins according to anatomical location and symptom status at presentation. Green boxes denote spontaneous passage, amber boxes intervention, and yellow boxes minor complications. Faded boxes indicate subgroups with no patients.

**Figure 3:**
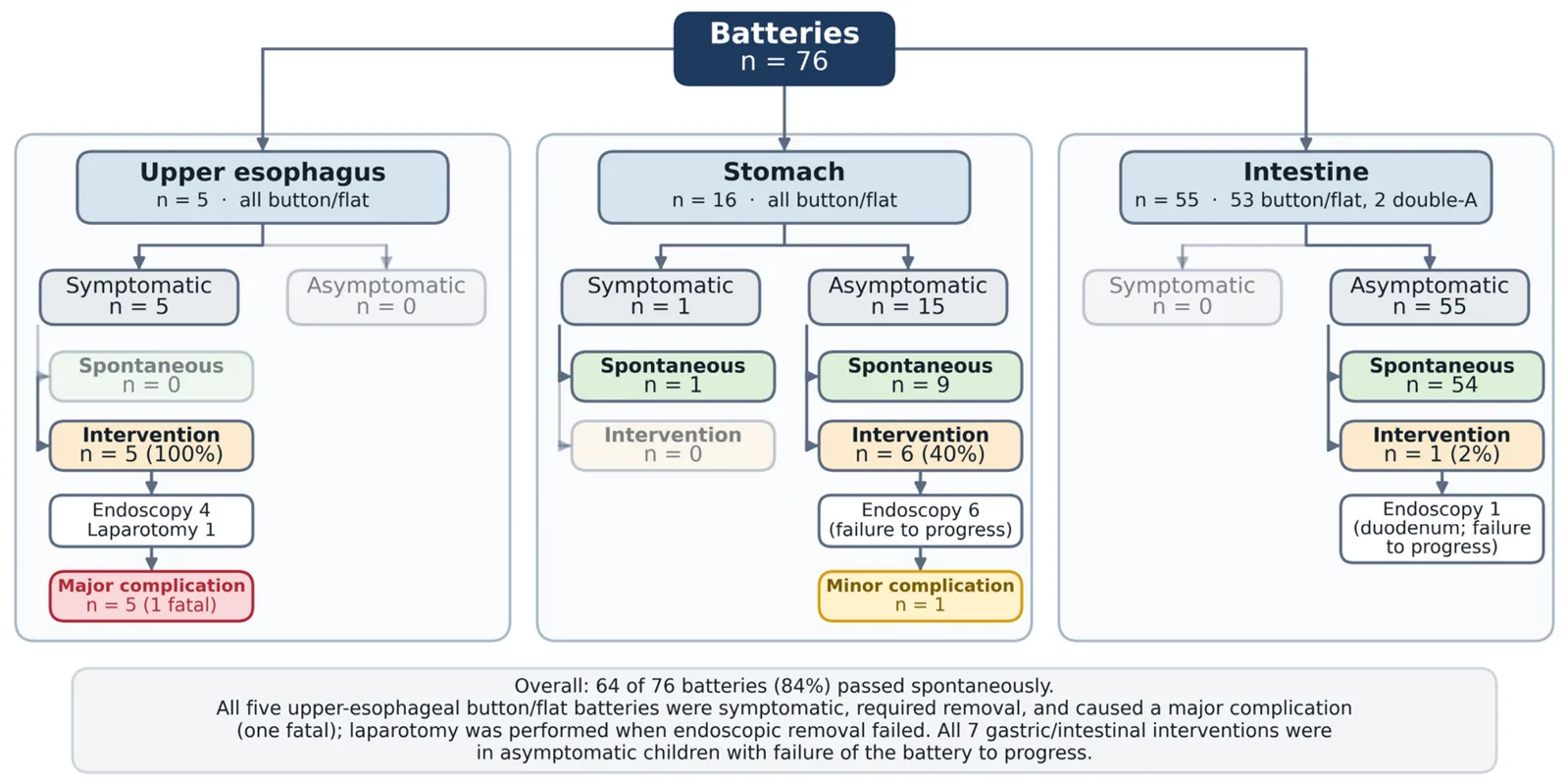
Management approach and outcomes for ingested batteries according to anatomical location and symptom status at presentation. Green boxes denote spontaneous passage, amber boxes intervention, red boxes major complications, and yellow boxes minor complications. Faded boxes indicate subgroups with no patients.

**Figure 4:**
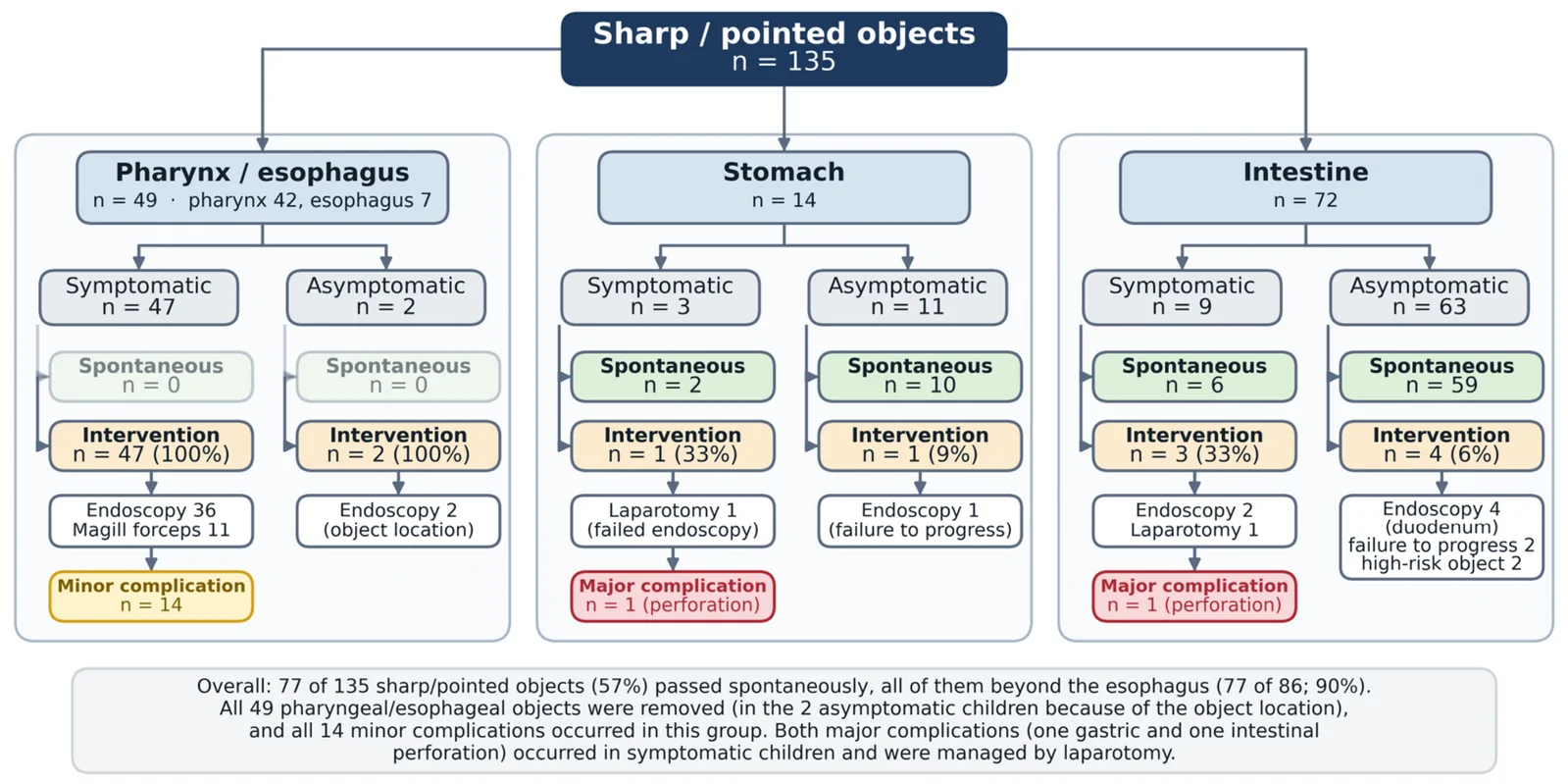
Management approach and outcomes for ingested sharp/pointed objects according to anatomical location and symptom status at presentation. Green boxes denote spontaneous passage, amber boxes intervention, red boxes major complications, and yellow boxes minor complications. Faded boxes indicate subgroups with no patients.

**Figure 5:**
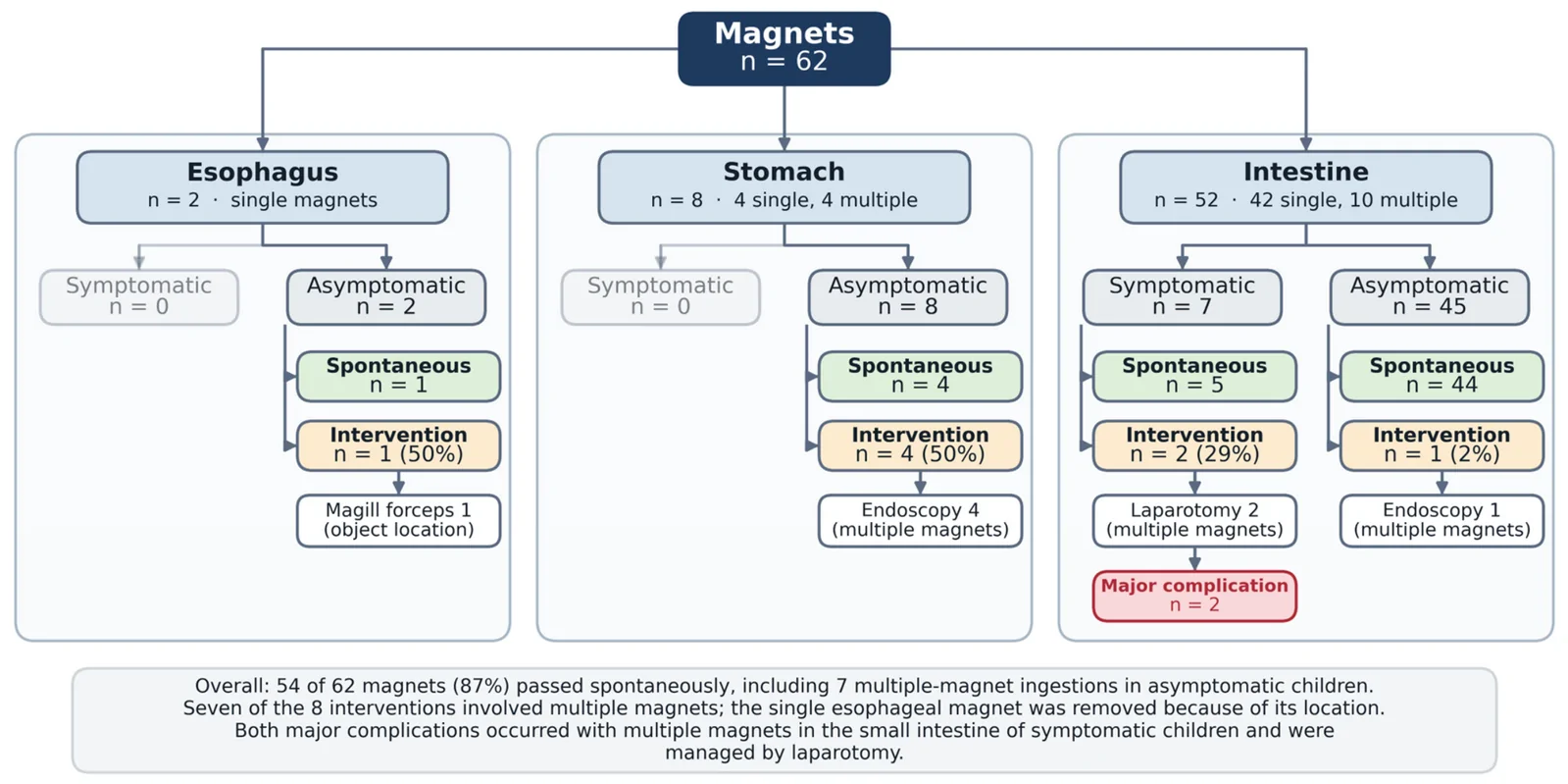
Management approach and outcomes for ingested magnets according to anatomical location and symptom status at presentation. Green boxes denote spontaneous passage, amber boxes intervention, red boxes major complications, and yellow boxes minor complications. Faded boxes indicate subgroups with no patients.

Foreign body ingestion was confirmed by plain radiography in 702 children, and by direct visualization on oropharyngeal examination or during esophagoscopic examination in 52 children. Most of the children were asymptomatic at presentation (581; 77.1%). Among the 173 symptomatic children (22.9%), the most frequent findings were dysphagia/odynophagia (100; 13.3%), vomiting (40; 5.3%), and gagging/feeding refusal and increased salivation (26; 3.4% each). The FB passed spontaneously in 572 cases (75.9%), while an interventional procedure was performed in 182 cases (24.1%). The most frequently performed procedure was endoscopy (136; 18.0%), followed by Magill forceps extraction (31; 4.1%), Foley catheter retrieval (10; 1.3%), and laparotomy (5; 0.7%). Of the 173 symptomatic children, 118 (68.2%) underwent an intervention, compared with 64 of the 581 asymptomatic children (11.0%). In these asymptomatic children, the recorded indication was a high-risk object or location in 47 cases (most commonly an esophageal coin, *n* = 33) and failure of the object to progress on follow-up radiographs in 17 cases. These 64 interventions involved coins (*n* = 37), batteries (*n* = 7), sharp/pointed objects (*n* = 7), magnets (*n* = 6), and other objects (*n* = 7); the indications for the first four groups are shown in Figure 2, Figure 3, Figure 4 and Figure 5. Overall, 235 children (31.2%) re-presented to our institution mostly for scheduled follow-up. Among these, 15 (6.4%) underwent further investigation, including endoscopy, for probable long-term complications like esophageal strictures.

Complications occurred in 29 children (3.8%); 20 of these (2.7%) were minor, and 9 (1.2%) were major. The specific diagnosis, diagnostic method, and timing of diagnosis for each complication are provided in Table S1. All complications occurred in children who underwent an interventional procedure; none was recorded among the 572 children managed conservatively; however, because these children did not undergo endoscopic examination, minor mucosal injuries could not be assessed in this group. All acute mucosal and transmural injuries (mucosal injury, burn, necrosis, and perforation) were identified at the time of the interventional procedure, whereas mediastinitis, delayed hemorrhage due to aorto-esophageal fistula, and esophageal stricture became apparent during hospitalization or follow-up. No procedure-related (iatrogenic) complications were recorded; all 29 complications were attributed to the foreign body itself. The highest complication rates were observed with food boluses (3/8; 37.5%), sharp/pointed objects (16/135; 11.9%), and batteries (6/76; 7.9%), whereas coins, the most frequently ingested object, had the lowest complication rate (1/315; 0.3%) (Table 2).

### 3.3. Factors Associated with Complications

In univariate analysis, age was not significantly associated with complications. In contrast, symptomatic presentation was strongly associated with the development of complications, but this association reflected near-complete separation (28 of the 29 complications occurred in symptomatic children; OR 112.0, 95% CI 15.1–830.0). A sharp/pointed object (OR 6.3, 95% CI 2.9–13.4) was associated with higher odds of complications, whereas ingestion of coins (OR 0.05, 95% CI 0.006–0.4) and other blunt objects (OR 0.1, 95% CI 0.02–0.9) were associated with lower odds. Food-bolus impaction was infrequent (*n* = 8); all underwent intervention, and three developed minor complications (none had serious complications). Due to the small number of cases, the corresponding OR (Table 3) is unstable and was not interpreted further. Battery ingestion was not significantly associated with complications (OR 2.44, 95% CI 0.96–6.20). All battery-related complications occurred with button/flat batteries; the two cylindrical (double-A) batteries were located in the colon of asymptomatic children and passed spontaneously without complications, and excluding them yielded a similar estimate (OR 2.51, 95% CI 0.99–6.38). Pharyngeal or esophageal localization was associated with markedly higher odds (combined OR 17.5, 95% CI 6.6–46.6), whereas intestinal localization was associated with lower odds of complications (OR 0.06, 95% CI 0.02–0.2). In the parsimonious multivariable model, vomiting (OR 5.7, 95% CI 1.9–16.5; *p* = 0.002), a sharp/pointed object (OR 6.3, 95% CI 2.7–15.1; *p* < 0.001), and a pharyngeal or esophageal location (OR 12.6, 95% CI 4.6–34.2; *p* < 0.001) were each independently associated with the development of complications (Table 3). No collinearity was detected among these covariates (variance inflation factors, 1.02–1.06). A penalized (Firth) logistic regression, fitted because of the low event count, yielded closely similar estimates (vomiting OR 5.65, 95% CI 2.02–15.80; sharp/pointed OR 6.13, 2.65–14.15; pharyngeal/esophageal OR 11.47, 4.45–29.59). Model fit was adequate (Hosmer–Lemeshow χ^2^ = 1.62, df = 2, *p* = 0.45; apparent c-statistic 0.872, 95% CI 0.796–0.947), and internal validation by bootstrap resampling (1000 replicates) gave an optimism-corrected c-statistic of 0.866. In a sensitivity analysis using dysphagia/odynophagia as a variable instead of vomiting, dysphagia/odynophagia (OR 3.71, 95% CI 1.25–11.04; *p* = 0.018), a sharp/pointed object (OR 3.06, 95% CI 1.30–7.22; *p* = 0.011), and a pharyngeal or esophageal location (OR 6.69, 95% CI 1.95–22.98; *p* = 0.003) all remained associated with complications; the attenuation of the location estimate reflects the partial overlap between local esophageal symptoms and an esophageal location.

### 3.4. Major Complications

Major complications were rare (9 children; 1.2%) but clustered in a small number of object and location combinations. Five of the nine major complications were caused by a button/flat battery lodged in the upper part of the esophagus; one of these children, a 1.4-year-old girl, died of an aorto-esophageal fistula with massive hemorrhage. Two major complications were caused by multiple magnets in the small intestine, and two by a sharp/pointed object (one gastric, one intestinal). No major complications arose from a coin or other blunt object reaching the stomach or beyond. The individual characteristics of the nine children who developed a major complication, including age, FB type, location, presenting findings, the specific complication, and the intervention performed, are detailed in Table 4. Given the small number of events, these findings are descriptive and do not provide reliable comparative risk estimates for individual object types or locations.

### 3.5. Management According to Foreign Body Type and Location

The most common FBs requiring intervention were food bolus (100%), sharp/pointed objects (43%), and coins (22.5%) (Table 2). Coins, the most frequently ingested object, illustrate a dissociation between the need for intervention and the risk of complications. Despite its relatively higher removal rate, particularly in the esophagus, complications due to coin ingestion were extremely low (1/315; 0.3%, a single minor mucosal injury). In contrast, button/flat batteries lodged in the esophagus accounted for most major complications. The indication for the two asymptomatic intestinal interventions for a coin and a battery was non-progression of duodenal FBs. The management approaches applied, and the corresponding outcomes according to FB type and anatomical location, are summarized in Figure 2, Figure 3, Figure 4 and Figure 5 for coins, batteries, sharp/pointed objects, and magnets, respectively.

## 4. Discussion

In this large single-center cohort of 754 children with FB ingestion, most ingestions were asymptomatic. Overall, three-quarters of FBs were expelled spontaneously without intervention, and only 3.8% of children developed a complication, fewer than half of which were major. Major complications were concentrated in a small number of high-risk object–location combinations. Vomiting, sharp or pointed objects, and pharyngeal or esophageal location were independently associated with the development of complications in the multivariable model; given the interrelation of these features and the small number of events, these associations should be interpreted with caution. To our knowledge, this is among the largest single-center pediatric cohorts specifically evaluating the clinical factors and FB characteristics associated with complications following FB ingestion.

Our finding that coins are the most frequently swallowed object and that young children (1–5 years) are the majority is consistent with the epidemiology of pediatric FB ingestion across diverse geographic settings [8,10,17]. The high rate of spontaneous passage (75.9%) is also consistent with previous reports showing that most objects pass through the gastrointestinal tract uneventfully and that careful observation is appropriate for the majority of children [3,4,14]. Therefore, the key for physicians providing emergency care is not to intervene indiscriminately, but to selectively identify the at-risk minority.

We observed that the presence of any symptoms consistent with FB ingestion upon admission to the ED was very strongly associated with the development of complications: 28 out of 29 complications occurred in symptomatic children. Because this near-complete separation precludes a stable regression estimate, the binary “symptomatic” variable was not included in the multivariable model; nonetheless, it emphasizes its value as the first triage step in clinical examination. Among individual symptoms, vomiting was associated with approximately six-fold higher odds of complications; however, the wide CI (1.94–16.47) indicates considerable imprecision. These findings are in keeping with previous reports identifying symptoms at presentation as one of the key determinants of complicated outcomes [6,7,13].

Anatomical location was a second major determinant of complication risk in our study. Pharyngeal or esophageal placement showed the strongest association, with approximately 13-fold higher odds of complications (95% CI 4.61–34.24). In the sensitivity analysis, this estimate was attenuated when dysphagia/odynophagia was substituted for vomiting (OR 6.69), illustrating the close relationship between local esophageal symptoms and an upper gastrointestinal location. This is consistent with the principle that esophageal FBs, particularly those above the upper esophageal sphincter, have a high potential for mucosal damage and require urgent intervention [1,4,6]. In contrast, we observed that objects reaching the intestine were associated with significantly lower odds of complications; this situation generally supports a conservative, observational approach when an object passes beyond the duodenum [3,14,18].

Among FB types, a sharp/pointed object was associated with approximately six-fold higher odds of complications; this is consistent with previous pediatric data identifying such objects as high-risk [9,19]. Although complications occurred in 3 out of the 8 children with food-bolus impaction, this subgroup was too small for a reliable estimate. Food impaction is nevertheless clinically significant and should be managed like other upper gastrointestinal tract FBs. The apparent association of coin and other blunt-object ingestions with lower odds of complications can be explained by the benign nature of these objects, which overwhelmingly pass without incident [1,4]. The paradox of very low complication risk despite relatively frequent intervention in our cohort is consistent with recent data from a comparable-sized cohort in which coin ingestion, although rarely complicated, is independently associated with the need for endoscopic removal, especially for esophageal coins [20]. Thus, a low risk of complications should not be equated with a low need for intervention.

Although the small number of major complications (*n* = 9) prevents reliable comparative risk estimates, their distribution carries an important clinical message and is consistent with the known hazards of these objects. Five of the nine major complications were caused by a button/flat battery lodged in the upper esophagus. Of the 76 batteries in the cohort, 74 were button/flat batteries, and 2 were cylindrical (double-A) batteries, which generally carry a lower corrosive risk. Both cylindrical batteries passed spontaneously without complications, so all battery-related complications in our cohort occurred with button/flat batteries. Button batteries are uniquely dangerous because they can cause liquefaction necrosis within hours through local current flow and hydroxide production, leading to burns, strictures, perforation, fistulas, and mediastinitis [21,22]. In our cohort, one of these batteries caused esophageal burn and necrosis complicated by an aorto-esophageal fistula with fatal hemorrhage, highlighting the lethal potential of this injury. Our data reinforce the guideline recommendations that a button battery in the esophagus is a true emergency requiring immediate removal regardless of symptoms [11,12]. Two more major complications occurred due to multiple magnets in the small intestine. Multiple magnets can attract each other along the folds of the intestine and cause pressure necrosis, fistula, obstruction, or perforation; therefore, early intervention is recommended [10,23,24]. Our findings support the immediate removal of endoscopically accessible multiple magnets and close observation with a low surgical threshold for those beyond endoscopic access [23,25]. The remaining two major complications involved sharp/pointed objects in the stomach and intestine, further highlighting the high risk associated with this type of object. Therefore, these results suggest that the likelihood of complications depends not only on the anatomical location requiring intervention, but also on the inherent danger of the object itself.

Taken together, our findings allow a practical synthesis for physicians providing emergency care. In this cohort, complications occurred almost exclusively in symptomatic children. Vomiting, a sharp/pointed object, and a pharyngeal or esophageal location were independently associated with complications, and esophageal button batteries, multiple magnets, and sharp/pointed objects accounted for all major complications. Because object type, anatomical location, and presenting symptoms are inherently interrelated, these should be interpreted as statistically independent associations rather than independent biological effects; moreover, our study identifies associations and does not validate a clinical decision rule, which would require prospective evaluation. Nevertheless, these findings are consistent with existing risk-stratification algorithms [10,12,14] and with current ESPGHAN/NASPGHAN guidance [11,12]. Notably, the recent ESPGHAN position paper identifies the type of object, its anatomical location, the presence and severity of symptoms, and the time since ingestion as the key variables guiding management [26]; three of these correspond to the domains of our multivariable model. Consistent with these recommendations, our findings support rapid radiographic localization of a suspected radiopaque object, prompt removal of esophageal button batteries even in asymptomatic children, and urgent endoscopic removal of multiple magnets and sharp/pointed objects within endoscopic reach, along with close clinical and radiographic monitoring and establishing a low threshold for surgery when they are beyond reach. Coins and other blunt objects retained in the esophagus should also be removed, whereas for other asymptomatic objects that have passed the esophagus, the high rate of spontaneous passage supports conservative management with observation. Complications may be further reduced at two levels. First, rapid identification of high-risk object–location combinations allows for removal before mucosal damage progresses, which is particularly critical for button batteries, where burns can develop within hours. Second, many of these injuries can be prevented at the source through childproof battery compartments, safe storage of button batteries and small magnets, and caregiver awareness.

This study has several limitations. Its retrospective, single-center design carries a risk of selection and information bias; some clinical details may have been omitted from the record, and external validation in other settings would strengthen the generalizability of the findings. Complications were relatively infrequent (29 events); therefore, to maintain a sufficient number of events per variable and avoid overfitting, we deliberately limited the multivariable model to one non-redundant variable per domain. While this approach provides stable and internally consistent estimates, it excludes some clinically relevant variables, and the wide CIs for several variables reflect the limited number of events; the reported ORs should therefore be interpreted with caution. In addition, the model addressed all types of complications; major complications (*n* = 9) were too few to be modeled separately. Because mucosal injury can be detected only when the object is directly visualized, complications were identified exclusively in children who underwent an interventional procedure, and minor mucosal injuries in children managed conservatively may have gone undetected. Accordingly, the absence of recorded complications in this group indicates the absence of clinically apparent complications rather than of any mucosal injury. Likewise, re-presentations were identified from our institutional records, so delayed complications in children who presented to other centers may have been missed. Finally, because inclusion required confirmation of ingestion, radiolucent objects that had spontaneously passed through the gastrointestinal tract without any symptoms or intervention may be underrepresented, which could bias the case mix toward radiopaque foreign bodies. Despite these limitations, the large sample size and the systematic review of subsequent records for all children are important strengths.

## 5. Conclusions

Our findings indicate that most pediatric FB ingestions are asymptomatic and resolve spontaneously without intervention. The presence of vomiting, sharp or pointed objects, and foreign bodies lodged in the pharynx or esophagus were associated with complications in this cohort; because these features are interrelated and the number of events was small, they should be regarded as warning signs that warrant closer evaluation rather than as a validated clinical prediction rule. A button battery lodged in the esophagus should be considered a medical emergency requiring immediate removal, and the ingestion of multiple magnets necessitates either urgent endoscopic removal or very close monitoring. Recognizing these high-risk clinical scenarios may help emergency physicians identify children who warrant closer evaluation and prioritize specialist assessment.

Importantly, the likelihood of intervention and the risk of serious complications are not synonymous. Although commonly ingested objects such as coins frequently require removal when retained in the esophagus, they are generally associated with a very low risk of severe harm compared with intrinsically hazardous objects such as button batteries. Thus, risk stratification should integrate both the anatomical location and the inherent characteristics of the ingested object to guide timely and appropriate management.

## Figures and Tables

**Table 1:** Demographic and foreign body characteristics of the patients (N = 754).

Variable	*n* (%)
Sex, male	434 (57.6)
Age, years ^†^	3.6 (2.2–5.7)
Age group, years	
<1	44 (5.8)
1–5	534 (70.8)
6–9	111 (14.7)
10–18	65 (8.6)
Comorbidity	
Neuromotor disorder	11 (1.5)
Behavioural disorder	6 (0.8)
Anatomic abnormality	4 (0.5)
Foreign body type	
Coin	315 (41.8)
Sharp/pointed object	135 (17.9)
Battery	76 (10.1)
button/flat	74 (9.8)
double-A	2 (0.3)
Magnet	62 (8.2)
single	48 (6.4)
multiple	14 (1.9)
Food bolus	8 (1.1)
Other	158 (21.0)
Foreign body location	
Pharynx	63 (8.4)
Upper esophagus	86 (11.4)
Mid esophagus	6 (0.8)
Lower esophagus	25 (3.3)
Stomach	81 (10.7)
Duodenum	45 (6.0)
Jejunum–ileum	230 (30.5)
Colon	218 (28.9)

^†^ Median (interquartile range).

**Table 2:** Clinical findings and outcomes of the patients.

Variable	*n* (%)	
Symptom/sign		
Absent	581 (77.1)	
Present	173 (22.9)	
Symptoms		
Dysphagia/odynophagia	100 (13.3)	
Vomiting	40 (5.3)	
Gagging/feeding refusal	26 (3.4)	
Increased salivation	26 (3.4)	
Abdominal pain	22 (2.9)	
Cough	22 (2.9)	
Treatment		
Spontaneous passage	572 (75.9)	
Interventional procedure	182 (24.1)	
Interventional procedures		
Endoscopy	136 (18.0)	
Magill forceps	31 (4.1)	
Foley catheter	10 (1.3)	
Laparotomy	5 (0.7)	
Complication *	29 (3.8)	
Minor	20 (2.7)	
Major	9 (1.2)	
By foreign body type		
	Intervention **	Complication **
Food bolus	8/8 (100.0)	3/8 (37.5)
Sharp/pointed object	58/135 (43.0)	16/135 (11.9)
Coin	71/315 (22.5)	1/315 (0.3)
Other	25/158 (15.8)	1/158 (0.6)
Battery	12/76 (15.8)	6/76 (7.9)
Magnet	8/62 (12.9)	2/62 (3.2)

* Minor: local mucosal injury (mucosal hyperemia, edema, erosion, or bleeding). Major: esophageal burn/stricture/perforation/fistula, mediastinitis, necrosis, gastrointestinal hemorrhage, or intestinal obstruction/adhesion/perforation. ** Expressed as the number of interventions/complications per number of ingestions of each foreign body type.

**Table 3:** Univariate and multivariable analysis of factors associated with the development of complications.

	Univariate		Multivariable	
	OR (95% CI)	*p*	OR (95% CI)	*p*
Age > 5 years ^†^	2.07 (0.96–4.48)	0.063		
Symptomatic ^‡ §^	112.00 (15.11–830.01)	<0.001 **		
Vomiting	6.67 (2.66–16.73)	<0.001 **	5.65 (1.94–16.47)	0.002 **
Abdominal pain	6.28 (1.98–19.94)	0.002 **		
Dysphagia/odynophagia	17.92 (7.89–40.69)	<0.001 **		
Gagging/feeding refusal	5.11 (1.64–15.95)	0.005 **		
Salivation	18.74 (7.45–47.13)	<0.001 **		
Cough	2.61 (0.58–11.74)	0.211		
Foreign body type ^‡^				
Food bolus ^‖^	16.62 (3.77–73.27)	<0.001 **		
Sharp/pointed object	6.27 (2.94–13.37)	<0.001 **	6.34 (2.66–15.07)	<0.001 **
Magnet	0.82 (0.19–3.54)	0.791		
Battery	2.44 (0.96–6.20)	0.060		
Coin	0.047 (0.006–0.35)	0.003 **		
Other blunt objects	0.13 (0.017–0.96)	0.045 *		
Anatomical location ^‡^				
Pharynx	7.91 (3.55–17.63)	<0.001 **		
Esophagus	4.85 (2.27–10.38)	<0.001 **		
Stomach	0.61 (0.14–2.60)	0.500		
Intestinal	0.056 (0.017–0.19)	<0.001 **		
Pharynx or esophagus (combined)	17.51 (6.57–46.63)	<0.001 **	12.57 (4.61–34.24)	<0.001 **

OR: odds ratio, CI: confidence interval. ^†^ Age dichotomized as >5 vs. ≤5 years; ^‡^ present vs. absent (reference: all other patients). The multivariable model was restricted to one non-redundant variable per domain to maintain ≈ 10 outcome events per variable. ^§^ “Symptomatic” was not entered into the multivariable model because of near-complete separation (28/29 complications were symptomatic). ^‖^ This estimate, derived from eight children in whom three developed complications, is unstable and is presented for the purpose of providing complete information. * *p* < 0.05, ** *p* < 0.01.

**Table 4:** Characteristics of the patients who developed a major complication.

Patient	Age (Years)	Sex	FB Type	Location	Clinical Findings	Type of Complication	Intervention
1	2.8	F	Button/flat battery	Upper esophagus	Vomiting, dysphagia	Esophageal burn	Endoscopy
2	1.2	M	Button/flat battery	Upper esophagus	Dysphagia, hypersalivation	Esophageal burn	Endoscopy
3	0.9	F	Button/flat battery	Upper esophagus	Vomiting, dysphagia, hypersalivation	Esophageal burn, stricture	Laparotomy
4	1.0	M	Button/flat battery	Upper esophagus	Dysphagia, gagging/feeding refusal, hypersalivation	Esophageal burn, mediastinitis	Endoscopy
5	1.4	F	Button/flat battery	Upper esophagus	Vomiting, dysphagia, hypersalivation, gagging/feeding refusal, cough	Esophageal burn, necrosis, aorto-esophageal fistula, abundant bleeding, death	Endoscopy
6	6.3	F	Multiple magnets	Jejunum-ileum	Abdominal pain	Mucosal penetration, intestinal adhesion	Laparotomy
7	2.8	M	Multiple magnets	Jejunum-ileum	Abdominal pain	Duodenal erosion, necrosis, intestinal adhesion	Laparotomy
8	2.6	M	Sharp/pointed object	Jejunum-ileum	Vomiting, abdominal pain	Intestinal perforation	Laparotomy
9	16.1	F	Sharp/pointed object	Stomach	Abdominal pain	Gastric perforation	Laparotomy

FB: foreign body, F: female, M: male. Patients are grouped by foreign body type (button/flat batteries, *n* = 5; multiple magnets, *n* = 2; sharp/pointed objects, *n* = 2). In Patient 3, after an unsuccessful attempt with endoscopic removal, the battery was advanced into the stomach and retrieved via an anterior gastrotomy through an upper midline mini-laparotomy.

## Data Availability

The data presented in this study are available from the corresponding author upon reasonable request. The data are not publicly available due to privacy and ethical restrictions concerning pediatric patient information.

## Supplementary Materials

The following supporting information can be downloaded at: https://www.mdpi.com/article/10.3390/children13101381/s1, Table S1: Specific diagnosis, diagnostic method, and timing of diagnosis for each complication.

## Abbreviations

The following abbreviations are used in this manuscript:

FB	Foreign body
ED	Emergency department
GI	Gastrointestinal
ESPGHAN	European Society for Paediatric Gastroenterology, Hepatology and Nutrition
NASPGHAN	North American Society for Pediatric Gastroenterology, Hepatology, and Nutrition
φ	Phi
OR	Odds ratio
CI	Confidence interval
SPSS	Statistical Package for the Social Sciences
STROBE	Strengthening the Reporting of Observational Studies in Epidemiology
