# Supplementary material for: Factors Associated with Complications in Pediatric Foreign Body Ingestion: A Retrospective Cohort Study of 754 Children
Source: Children (Basel). 2026 Oct 12;13(10):1381. doi: 10.3390/children13101381 (PMC13703468; doi:10.3390/children13101381)
Supplement: Supplementary file 1 [file children-13-01381-s001.zip › children-4586470-supplementary.pdf]

Table S1. Specific diagnosis, diagnostic method, and timing of diagnosis for each complication.

No.	Foreign body type	Location	Complication	Diagnosed by	Timing of diagnosis
Major complications (patients numbered as in Table 4)					
1	Button/flat battery	Upper esophagus	Esophageal burn	Endoscopy	Interventional procedure
2	Button/flat battery	Upper esophagus	Esophageal burn	Endoscopy	Interventional procedure
3	Button/flat battery	Upper esophagus	Esophageal burn	Endoscopy	Interventional procedure
			Esophageal stricture	Follow-up endoscopy	Follow-up
4	Button/flat battery	Upper esophagus	Esophageal burn	Endoscopy	Interventional procedure
			Mediastinitis	CT	After the interventional procedure
5	Button/flat battery	Upper esophagus	Esophageal burn and necrosis	Endoscopy	Interventional procedure
			Aorto-esophageal fistula	CT angiography	After the interventional procedure
			Massive hemorrhage (fatal)	Clinical	After the interventional procedure
6	Multiple magnets	Jejunum–ileum	Mucosal penetration, intestinal adhesion	Intraoperative findings (laparotomy)	Interventional procedure
7	Multiple magnets	Jejunum–ileum	Duodenal erosion, necrosis, intestinal adhesion	Intraoperative findings (laparotomy)	Interventional procedure
8	Sharp/pointed object	Jejunum–ileum	Intestinal perforation	Intraoperative findings (laparotomy)	Interventional procedure
9	Sharp/pointed object	Stomach	Gastric perforation	Intraoperative findings (laparotomy)	Interventional procedure
Minor complications					
10	Button/flat battery	Stomach	Mucosal erosion	Endoscopy	Interventional procedure
11	Coin	Upper esophagus	Mucosal hyperemia and edema	Endoscopy	Interventional procedure
12	Food bolus	Lower esophagus	Mucosal erosion	Endoscopy	Interventional procedure
13	Food bolus	Mid esophagus	Mucosal erosion with edema and fibrin	Endoscopy	Interventional procedure
14	Food bolus	Upper esophagus	Mucosal hyperemia and edema	Endoscopy	Interventional procedure
15	Other	Upper esophagus	Mucosal erosion with fibrin	Endoscopy	Interventional procedure
16	Sharp/pointed object	Mid esophagus	Mucosal erosion	Endoscopy	Interventional procedure
17	Sharp/pointed object	Pharynx	Mucosal hyperemia at the penetration site	Endoscopy	Interventional procedure

No.	Foreign body type	Location	Complication	Diagnosed by	Timing of diagnosis
18	Sharp/pointed object	Pharynx	Mucosal edema and hyperemia at the penetration site	Direct visualization (Magill forceps)	Interventional procedure
19	Sharp/pointed object	Pharynx	Mucosal hyperemia at the penetration site	Endoscopy	Interventional procedure
20	Sharp/pointed object	Pharynx	Mucosal bleeding at the penetration site	Endoscopy	Interventional procedure
21	Sharp/pointed object	Pharynx	Mucosal hyperemia at the penetration site	Endoscopy	Interventional procedure
22	Sharp/pointed object	Pharynx	Mucosal hyperemia at the penetration site	Endoscopy	Interventional procedure
23	Sharp/pointed object	Pharynx	Mucosal hyperemia at the penetration site	Endoscopy	Interventional procedure
24	Sharp/pointed object	Pharynx	Mucosal hyperemia at the penetration site	Endoscopy	Interventional procedure
25	Sharp/pointed object	Pharynx	Mucosal hyperemia at the penetration site	Endoscopy	Interventional procedure
26	Sharp/pointed object	Pharynx	Mucosal edema and hyperemia at the penetration site	Direct visualization (Magill forceps)	Interventional procedure
27	Sharp/pointed object	Pharynx	Mucosal hyperemia at the penetration site	Endoscopy	Interventional procedure
28	Sharp/pointed object	Upper esophagus	Mucosal erosion	Direct visualization (Magill forceps)	Interventional procedure
29	Sharp/pointed object	Upper esophagus	Mucosal erosion with bleeding	Direct visualization (Magill forceps)	Interventional procedure

CT: Computed tomography. Patients 1–9 correspond to the patients listed in Table 4. For patients with more than one complication, each complication is shown on a separate row with its own diagnostic method and timing.

Interventional procedure: the procedure performed to remove the foreign body (endoscopy, Magill forceps extraction, or laparotomy). Acute mucosal and transmural injuries were identified during the interventional procedure by endoscopy, by direct visualization during Magill forceps extraction, or from intraoperative findings; complications that became apparent later were diagnosed by follow-up endoscopy, CT, CT angiography, or clinical assessment. No procedure-related (iatrogenic) complication was recorded; all 29 complications were attributed to the foreign body itself.
